# Saikosaponin A mediates the inflammatory response by inhibiting the MAPK and NF-κB pathways in LPS-stimulated RAW 264.7 cells

**DOI:** 10.3892/etm.2013.988

**Published:** 2013-03-04

**Authors:** JIE ZHU, CHENGQUN LUO, PING WANG, QUANYONG HE, JIANDA ZHOU, HAO PENG

**Affiliations:** 1 Department of Burns and Plastic Surgery, Third Xiangya Hospital, Central South University, Changsha, Hunan 410013, P.R. China; 2 Centre of PET-CT, Hunan Provincial Tumor Hospital, Changsha, Hunan 410013, P.R. China

**Keywords:** saikosaponin A, inflammation, lipopolysaccharide, inflammatory cytokine, nuclear factor-κB, mitogen-activated protein kinase

## Abstract

Saikosaponin A (SSA) is a major triterpenoid saponin isolated from *Radix bupleuri* (RB), a widely used Chinese traditional medicine to treat various inflammation-related diseases. The aim of this study was to investigate the anti-inflammatory activity, as well as the molecular mechanism of SSA in lipopolysaccharide (LPS)-stimulated RAW 264.7 cells. In this study, we demonstrated that SSA markedly inhibits the expression of certain immune-related cytotoxic factors, including cyclooxygenase-2 (COX-2) and inducible nitric-oxide synthase (iNOS), as well as pro-inflammatory cytokines, including tumor necrosis factor (TNF)-α, interleukin (IL)-1β and IL-6. It also significantly upregulates the expression of IL-10, an important anti-inflammatory cytokine, suggesting its anti-inflammatory activity in LPS-stimulated macrophages. We further demonstrated that SSA inhibits the activation of the nuclear factor-κB (NF-κB) signaling pathway by suppressing the phosphorylation of inhibitory NF-κB inhibitor α (IκBα) and thus holding p65 NF-κB in the cytoplasm to prevent its translocation to the nucleus. In addition, SSA also inhibits the mitogen-activated protein kinase (MAPK) signaling pathway by downregulating the phosphorylation of p38 MAPK, c-Jun N-terminal kinase (c-JNK) and extracellular signal-regulated kinase (ERK), the three key components of the MAPK family. In conclusion, our study demonstrates that SSA has an anti-inflammatory effect by regulating inflammatory mediators and suppressing the MAPK and NF-κB signaling pathways in LPS-stimulated RAW 264.7 cells.

## Introduction

*Radix Bupleuri* (RB), isolated from the dried roots of *Bupleurum chinense* DC or *Bupleurum scorzonerifolium* Willd, has been used as a health product and natural remedy for centuries in traditional Chinese medicine, based on its hepato-protective, antipyretic, analgesic, immunomodulatory and anti-inflammatory effects (1,2). As major bioactive compounds isolated from RB, saikosaponins have numerous biological activities, including immunoregulatory, anti-inflammatory, anti-bacterial and anti-viral activity (3,4). One study demonstrated that saikosaponin A (SSA) exhibits anti-inflammatory activity (5). However, the potential molecular mechanism of SSA in terms of the anti-inflammatory signaling pathways has not been fully determined.

Inflammation is a beneficial host response to foreign challenge or tissue injury, helping facilitate the restoration of tissue structure. However, prolonged inflammation is not beneficial as it contributes to the pathology of a number of diseases (6,7). Therefore, anti-inflammatory agents have potential therapeutic effects for various inflammation-related diseases. It is well established that activated immunocytes are involved in the inflammation process, particularly macrophages, which play a crucial role in the specific and non-specific immune responses during inflammation (8). Lipopolysaccharide (LPS) induces the release of inflammatory mediators in macrophages, leading to the production of inducible nitric oxide synthase (iNOS), tumor necrosis factor (TNF)-α, interleukin (IL)-1β and IL-6 (9,10).

Cytokines play essential roles in the inflammatory response, mainly due to their crucial effects on the differentiation, maturation and activation of cells (11). However, excessive production of cytokines harms organisms (6). It has been reported that patients suffering from inflammatory diseases present abnormalities in pro- and anti-inflammatory cytokines (12). Inflammatory cytokine release in response to LPS is mediated by the activation of nuclear factor κ-light-chain enhancer of activated B cells (NF-κB) and mitogen-activated protein kinase (MAPK) (13,14). NF-κB is a family of transcription factors and regulates the expression of a number of immune-related cytotoxic factors, including iNOS and cyclooxygenase-2 (COX-2), and pro-inflammatory cytokines, including TNF-α, IL-1β, IL-6 and IL-8 (15,16). The MAPK family also induces the production of immune-related cytotoxic factors and pro-inflammatory cytokines (17,18). Therefore, NF-κB and MAPKs are well-recognized as targets of anti-inflammatory agents.

In the present study, we examined the effects of SSA on the production of various inflammatory cytokines in LPS-stimulated mouse RAW 264.7 macrophages. We also investigated its anti-inflammatory mechanism, focusing on inflammatory signaling pathways. To our knowledge, this is the first report demonstrating that SSA inhibits the production of immune-related cytotoxic factors and inflammatory cytokines induced by LPS by inhibiting the NF-κB and MAPK signaling pathways.

## Materials and methods

### Reagents

SSA was purchased from Sichuan Victory Biotechnology Co., Ltd. (Sichuan, China), with 98% purity detected by high performance liquid chromatography (HPLC). LPS (*Escherichia coli* 026:B6), dimethyl sulfoxide (DMSO) and 3-[4,5-dimethylthiazol- 2-yl]-2,5-diphenyltetrazolium bromide (MTT) were purchased from Sigma (St. Louis, MO, USA). TNF-α, IL-1β, IL-6 and IL-10 enzyme-linked immunosorbent assay (ELISA) kits were purchased from R&D Systems (Minneapolis, MN, USA). Dulbecco’s modified Eagle’s medium (DMEM) and fetal bovine serum (FBS) were purchased from HyClone Laboratories of Thermo Scientific (Logan, UT, USA).

The antibodies, including iNOS, COX-2, NF-κB (p65) and β-actin were obtained from Cayman Chemical Co. (Ann Arbor, MI, USA). Antibodies for phospho-extracellular signal-regulated kinases (ERK)1/2, ERK, phospho-p38, p38, phospho-Jun N-terminal kinase (JNK), JNK, IκBα and p65 were obtained from Cell Signaling Technology (Danvers, MA, USA).

### Cell culture and sample treatment

The mouse macrophage cell line RAW 264.7 was obtained from the Center of Cellular Resources, Central South University, Changsha, China. Cells were cultured in DMEM supplemented with 10% heat-inactivated FBS, 3 mM glutamine, 100 U/ml penicillin and 100 *μ*g/ml streptomycin at 37°C under a humidified atmosphere of 5% CO_2_. In all experiments, cells were left to acclimate for 24 h before treatment. SSA was added 1 h prior to LPS (1 mg/l) treatment. The study was approved by the ethics committee of Central South University, Changsha, China.

### MTT assay for cell viability

Cytotoxicity induced by SSA was analyzed by MTT assay. RAW 264.7 cells were plated at a density of 1×10^4^ cells/ml onto 96-well plates containing 100 *μ*l DMEM and incubated overnight. After acclimating for 24 h, the cells were treated with 100 *μ*l SSA at various concentrations (3.125, 6.25, 12.5, 25, 50 and 100 *μ*M) for 1 h, followed by stimulation with 50 *μ*l LPS (1 mg/l) for 18 h. Subsequently, 20 *μ*l MTT (5 mg/ml, 20 *μ*l/well) in FBS-free medium was added to each well and further incubated for 4 h. Cell-free supernatants were then removed and cells were resolved with 150 *μ*l DMSO per well, followed by optical density measurement at 490 nm with a ELX800-UV absorbance microplate reader (BioTek Instruments Inc., Winooski, VT, USA).

### Cytokine determination

To determine the effects of SSA on cytokine release in LPS-stimulated cells, the production of TNF-α, IL-1β, IL-6 and IL-10 was measured by ELISA. RAW 264.7 cells were grown in a 6-well plate at a density of 3×10^5^ cells/well for 24 h. The cells were pretreated with various concentrations of SSA compounds for 2 h and further challenged with LPS for an additional 18 h at 37°C with 5% CO_2_. The supernatants were then collected and centrifuged at 1,000 x g, 4°C for 10 min. The levels of TNF-α, IL-1β, IL-6 and IL-10 in the supernatants were determined using ELISA kits, according to the manufacturer’s instructions.

### Real-time fluorescent quantitative polymerase chain reaction (PCR)

RAW 264.7 cells (4×10^5^ cells/ml), cultured in 6-well plates for 24 h, were pretreated with various concentrations (3.125, 6.25 and 12.5 *μ*M) of SSA for 2 h before treatment with 1 *μ*g/ml LPS for 3 h in a 37°C, 5% CO_2_ incubator. Following two washes with ice-cold phosphate-buffered saline (PBS), the cells were harvested and total cellular RNA was isolated using the TRIzol reagent, according to the manufacturer’s instructions (Invitrogen Life Technologies, Carlsbad, CA, USA). For the real-time PCR, 1 *μ*g total RNA was reverse-transcribed to synthesize cDNA using a first-strand cDNA synthesis kit (Takara, Dalian, China). Quantitative real-time PCR was performed on a Bio-Rad CFX 96 real-time PCR detection system in a 30 ml reaction volume containing iQ™ SYBR-Green Supermix (Bio-Rad, Hercules, CA, USA), 100 nM primers and 1 ml appropriately diluted cDNA template. The parameters of the PCR reaction were as follows: 94°C for 3 min for one cycle, then 94°C for 30 sec, 55–59°C for 30 sec, 72°C for 45 sec for 30 cycles and 72°C for 5 min for one cycle. The relative gene expression was calculated by the comparative Ct method (2^−ΔΔCt^), using glyceraldehyde 3-phosphate dehydrogenase (GAPDH) as the house keeping gene. The primer sequences for analysis of TNF-α, IL-1β, IL-6 and GAPDH mRNA are presented in Table I.

### Western blot analysis

Western blot analysis was performed to evaluate the effect of the test compound on iNOS, COX-2, NF-κB (p65) and inhibitory NF-κB inhibitor α (IκBα) in the cytosol and nucleus, as well as the expressions of P38 MAPK, c-JNK and ERK. The RAW 264.7 cells were cultivated in a 6-well plate for 24 h and then received appropriate treatment with SSA in the absence or presence of LPS for 2 h. After treatment for 18 h with LPS, the cells were harvested and the total protein, cytosol protein and nuclear protein were extracted using a Nuclear-Cytosol Extraction Kit (Cell Signaling Technology). β-actin was used as the control. The protein was separated on polyacrylamide gels and then transferred onto a polyvinylidene fluoride (PVDF) membrane. The membranes were blocked and incubated with different antibodies, followed by incubation with the horseradish peroxidase (HRP)-linked secondary antibody. The signals were detected using an enhanced chemiluminescence (ECL) reagent (Bio-Rad). The images were quantified by Bio-Rad Quantity One software. The quantities of the target bands were normalized by β-actin.

### Statistical analysis

Data, expressed as means ± standard deviation, were analyzed by one-way analysis of variance (ANOVA). Significant differences were determined with Tukey’s multiple range tests. All tests were performed using SPSS 13.0 software (SPSS Inc., Chicago, IL, USA). P<0.05 was considered to indicate a statistically significant difference.

## Results

### Cytotoxicity of SSA on RAW 264.7 cells

**Figure 1:**
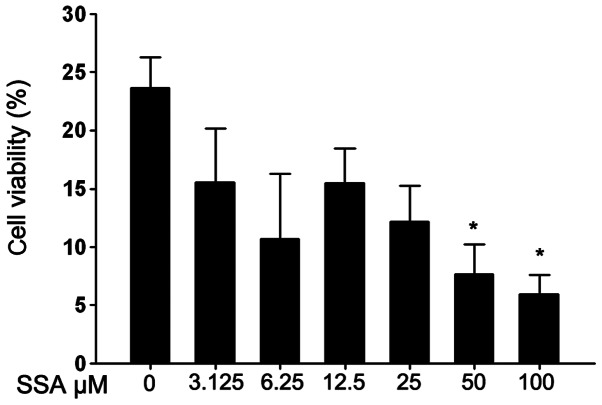
Effects of SSA on the viability of LPS-stimulated RAW 264.7 cells. Cells were treated with various concentrations of SSA. Cell viability was measured by MTT assay. Data were presented as mean ± SD from three separate experiments. ^*^P<0.05, compared to the control group. SSA, saikosaponin A; LPS, lipopolysaccharide; SD, standard deviation.

Prior to evaluating the anti-inflammatory activity of SSA, the cytotoxic effect of SSA on RAW 264.7 cells was tested using the MTT assay. As shown in Fig. 1, cell viability was significantly reduced with 12.5–100 *μ*M SSA, while 3.125 and 6.25 *μ*M SSA had no effect on LPS-stimulated RAW 264.7 cells.

### SSA inhibits the release of LPS-induced pro-inflammatory cytokines in RAW 264.7 cells

**Figure 2:**
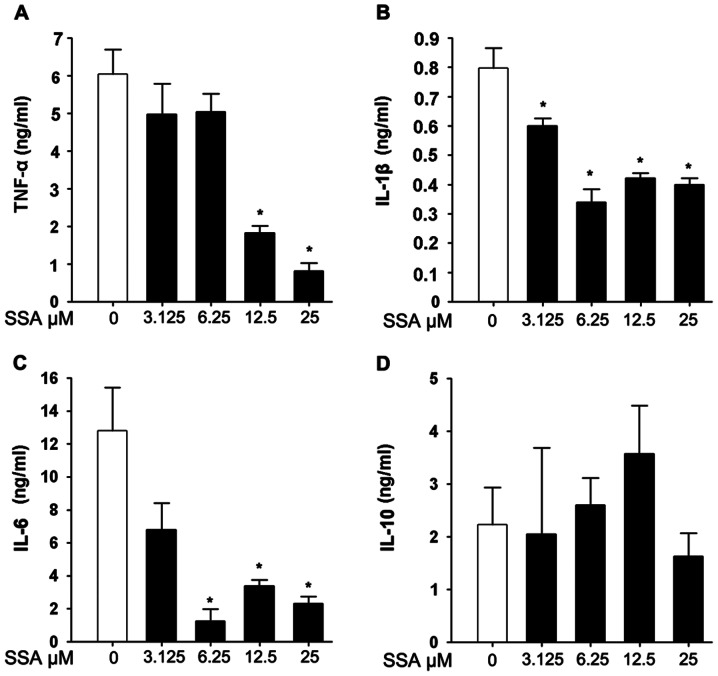
Effects of SSA on TNF-α (A), IL-1β (B), IL-6 (C) and IL-10 (D) expression in LPS-stimulated RAW 264.7 cells. Cells were treated with various concentrations of SSA. Data were derived from six independent experiments and presented as mean ± SD. ^*^P<0.05, compared to the control group. SSA, saikosaponin A; LPS, lipopolysaccharide; TNF, tumor necrosis factor; IL, interleukin; SD, standard deviation.

TNF-α, IL-1β, IL-6 and IL-10 concentrations in the culture supernatants of RAW 264.7 cells were evaluated by ELISA. As shown in Fig. 2A, TNF-α was significantly inhibited by pretreatment with SSA in a dose-dependent manner. A similar tendency was also observed in IL-6 and IL-1β production at various concentrations of SSA (Fig. 2B and C). However, SSA pretreatment had no significant effect on IL-10 compared to the control group in this assay (Fig. 2D).

### SSA inhibits the mRNA level of TNF-α, IL-1β, IL-6 and IL-10 in LPS-stimulated RAW 264.7 cells

**Figure 3:**
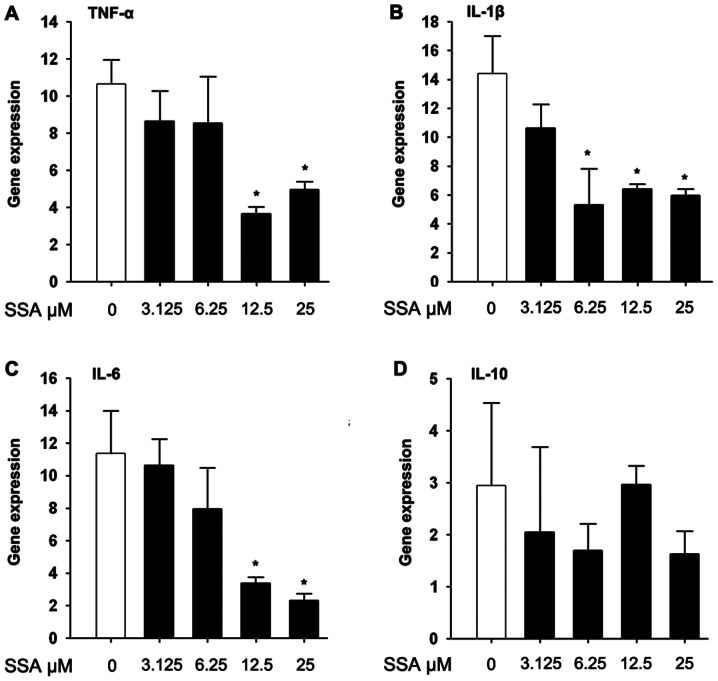
Inhibitory effects of SSA on TNF-α (A), IL-1β (B), IL-6 (C) and IL-10 (D) mRNA expression in LPS-stimulated RAW 264.7 cells. Cells were treated with various concentrations of SSA. Data are presented as mean ± SD from three separate experiments. ^*^P<0.05, compared to the control group. SSA, saikosaponin A; LPS, lipopolysaccharide; TNF, tumor necrosis factor; IL, interleukin; SD, standard deviation.

Real-time PCR was employed to quantitate TNF-α, IL-6, IL-1β and IL-10 gene expression from cDNA samples. For the mRNA expression of pro-inflammatory cytokines, SSA pretreatment for 1 h significantly inhibited the expression of TNF-α, IL-1β and IL-6 compared to the control group, and upregulated the expression of IL-10 (Fig. 3).

### SSA suppresses the expression of iNOS and COX-2 in LPS-stimulated RAW 264.7 cells

**Figure 4:**
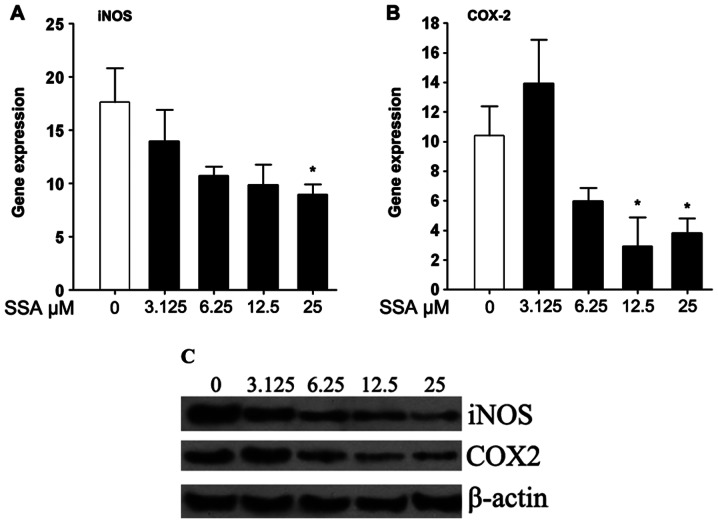
(A) Inhibitory effects of SSA on iNOS mRNA expression in LPS-stimulated RAW 264.7 cells. (B) Inhibitory effects of SSA on COX-2 mRNA expression in LPS-stimulated RAW 264.7 cells. (C) Inhibitory effects of SSA on iNOS and COX-2 protein expression in LPS-stimulated RAW 264.7 cells. Cells were treated with various concentrations of SSA. Data are presented as mean ± SD from three separate experiments. ^*^P<0.05, compared to the control group. SSA, saikosaponin A; iNOS, inducible nitric oxide synthase; LPS, lipopolysaccharide; COX, cyclooxygenase; SD, standard deviation.

Real-time PCR and western blotting were performed to determine the inhibitory effect of SSA on the mRNA and protein levels of iNOS and COX-2, respectively. As shown in Fig. 4A and B, the mRNA expressions of iNOS and COX-2 were reduced in a dose-dependent manner by SSA in LPS-stimulated RAW 264.7 cells. Also, SSA strongly downregulated iNOS and COX-2 protein expression (Fig. 4C).

### SSA suppresses the LPS-induced activation of NF-κB signaling in RAW 264.7 cells

**Figure 5:**
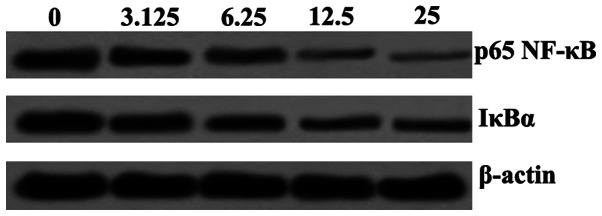
Inhibitory effects of SSA on the phosphorylation of p65 NF-κB and IκBα in LPS-stimulated RAW 264.7 cells. Cells were treated with various concentrations of SSA. SSA, saikosaponin A; NF, nuclear factor; LPS, lipopolysaccharide.

Western blotting was performed to determine the effect of SSA on LPS-induced NF-κB activation. The results revealed that p65 NF-κB and IκBα protein expression were downregulated by SSA. The p65 NF-κB and IκBα protein expression demonstrated a dose-dependent effect on suppression induced by SSA (Fig. 5).

### SSA suppresses the LPS-induced activation of MAPK signaling in RAW 264.7 cells

**Figure 6:**
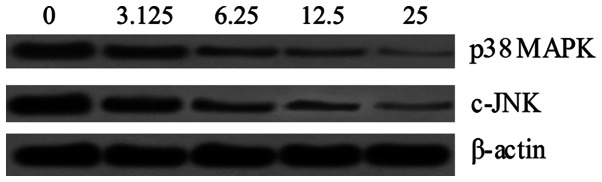
Inhibitory effects of SSA on the phosphorylation of p38 MAPK and c-JNK in LPS-stimulated RAW 264.7 cells. Cells were treated with various concentrations of SSA. SSA, saikosaponin A; MAPK, mitogen-activated protein kinase; c-JNK, c-Jun N-terminal kinase; LPS, lipopolysaccharide.

In order to understand the mechanism by which SSA inhibits LPS-induced production of inflammatory cytokines, we detected the possible connection between SSA and the MAPK pathway. Following SSA treatment, the phosphorylation of p38 MAPK and c-JNK had markedly decreased compared to the control in a dose-dependent manner (Fig. 6).

## Discussion

Macrophages play a crucial role in the specific and non-specific immune responses during the inflammatory process by producing a large amount of inflammatory mediators, including immune-related cytotoxic factors and inflammatory cytokines. Despite the beneficial effect during infection, excessive production of inflammatory mediators may cause edema, cellular metabolic stress and tissue necrosis (12). As a result, agents regulating inflammatory cytokines may have therapeutic effects. The present study demonstrated that LPS effectively induces the activation of macrophages, which is consistent with previous reports (19,20). By activating several signals and transcription factors, including MAPKs and NF-κB, LPS induces the activation of inflammatory cytokines in macrophages, leading to the production of TNF-α, IL-6, IL-1β and IL-10 (9,10). In the present study, we demonstrated that SSA markedly inhibits immune-related cytotoxic factors, including iNOS and COX-2, and pro-inflammatory cytokines, including TNF-α, IL-1β and IL-6. It also increased the protein and mRNA levels of the anti-inflammatory cytokine, IL-10, in LPS-stimulated RAW 264.7 macrophages. These data demonstrate the anti-inflammatory activity of SSA in macrophages stimulated by LPS.

To further clarify the molecular mechanism of the inhibitory effect of SSA on inflammatory mediators, we investigated the effects of SSA on the activation of two signaling pathways, NF-κB and MAPKs, in LPS-stimulated macrophages. LPS has been shown to induce the NF-κB signaling pathway in macrophages (21). NF-κB, a family of transcription factors, is universally expressed in various types of cells and regulates the transcription of a number of key inflammatory mediators, including COX-2, TNF-α, IL-1β, IL-6 and IL-10 (22). Therefore, the NF-κB signaling pathway acts as a core regulator of inflammation. Under normal conditions, NF-κB associates with IκBs, which sequester NF-κB in the cytoplasm. The activation of NF-κB begins with the phosphorylation of IκBα. Then, the phosphorylation of IκBα allows itself to be ubiquitinated and eventually degraded by the 26S proteasome (23). Once IκBα is degraded, the nuclear localization signal of NF-κB is not masked and NF-κB is able to translocate to the nucleus and promote the transcription of target genes (24). As demonstrated in the present study, in the control group, the phosphorylation levels of IκBα and p65 NF-κB were high following exposure to LPS; however, following administration of SSA, the phosphorylation of p65 NF-κB and IκBα were markedly decreased in a dose-dependent manner. These data indicate that SSA blocks the NF-κB signaling pathway by inhibiting the phosphorylation of IκBα, preventing NF-κB translocation to the nucleus.

The other major extracellular signaling pathway induced by inflammatory mediators is the MAPK pathway. In the MAPK family, p38 MAPK, c-JNK and ERKs are the most important components (18). LPS has been shown to induce the MAPK signaling pathway in macrophages (25), which is consistent with our data. In the present study, we identified that phosphorylation of p38 MAPK and c-JNK was high in LPS-stimulated macrophages; however, following administration of SSA, the phosphorylation of p38 MAPK and c-JNK significantly reduced in a dose-dependent manner, suggesting that the activation of the MAPK signaling pathway is inhibited by SSA. Since it is well established that MAPKs regulate various inflammatory mediators, including TNF, IL-1, IL-2, IL-6 COX-2 and iNOS (26–28), we consider that the anti-inflammatory activity of SSA is associated with its inhibitory effect on the MAPK signaling pathway.

In conclusion, this study demonstrated that SSA has an inhibitory effect on pro-inflammatory cytokines, as well as a facilitative effect on anti-inflammatory cytokines in LPS-stimulated macrophages. The mechanism of these actions involves the regulation of MAPK and NF-κB signals.

## Figures and Tables

**Table I:** Primers used for real-time PCR.

Gene	Primer	Sequence (5′-3′)
iNOS	Sense	CAAGCTGAACTTGAGCGAGGA
	Antisense	TTTACTCAGTGCCAGAAGCTGGA
COX-2	Sense	CTGGAACATGGACTCACTCAGTTTG
	Antisense	AGGCCTTTGCCACTGCTTGT
TNF-α	Sense	CCGCTCGTTGCCAATAGTGATG
	Antisense	CATGCCGTTGGCCAGGAGGG
IL-1β	Sense	GCACTACAGGCTCCGAGATGAA
	Antisense	GTCGTTGCTTGGTTCTCCTTGT
IL-6	Sense	CTTGGGACTGATGCTGGTGACA
	Antisense	GCCTCCGACTTGTGAAGTGGTA
IL-10	Sense	CGATGTTCTGTTCTGGTT
	Antisense	AAGACGCTTGACTTGAAG
GAPDH	Sense	AGTGGCAAAGTGGAGATT
	Antisense	GTGGAGTCATACTGGAACA

PCR, polymerase chain reaction; iNOS, inducible nitric oxide synthase; COX, cyclooxygenase; TNF, tumor necrosis factor; IL, interleukin; GAPDH, glyceraldehyde 3-phosphate dehydrogenase.
